# Assessing the impact of airborne particulate pollution on human skin utilizing a novel human skin equivalent containing MUTZ‐3‐derived Langerhans cells

**DOI:** 10.1002/btm2.10738

**Published:** 2024-12-13

**Authors:** Amy Simpson, Teresa DiColandrea, Stefan Przyborski

**Affiliations:** ^1^ Department of Biosciences Durham University Durham UK; ^2^ Procter & Gamble, Mason Business & Innovation Center Cincinnati Ohio USA; ^3^ Reprocell Europe Ltd Glasgow UK

**Keywords:** air pollution, diesel particulate matter, human skin model, inflammatory cytokines, Langerhans cells

## Abstract

Air pollution is an exogenous stressor known to have a detrimental impact on skin health through the induction of inflammation; however, the direct effect of topical pollution exposure is still being elucidated. Human skin equivalents (HSE) aim to reproduce in vitro the structure and function of the native skin tissue. However, HSEs typically lack skin‐resident immune cells, which could play a key role in the inflammatory response induced by pollution exposure. We outline the development of a HSE‐containing MUTZ‐3‐derived Langerhans cells (MUTZ‐3‐LCs), which show dendritic morphology and Langerhans cell marker expression. We demonstrated that HSE‐containing MUTZ‐3‐LC have lower basal levels of proinflammatory cytokines, but topical stimulation with allergens and irritant compounds induced a greater inflammatory response in these models compared to HSE without immune cells. To study the effect of pollution, we created a technique to apply diesel particulate matter (DPM) to HSEs. Though our microscopic analysis demonstrated that DPM does not penetrate the stratum corneum, we showed that DPM did induce production of proinflammatory cytokines, but notably only in HSEs containing MUTZ‐3‐LCs. These data suggest that topical exposure to air pollution can induce cutaneous inflammation and that skin‐resident immune cells contribute to this response. This highlights the significance of immune‐competent HSEs to the study of exogenous stressors in vitro.

**Translational Impact Statement**

We have created three‐dimensional skin tissue models in vitro containing Langerhans cells that show a proinflammatory response to particulate matter, a component of air pollution. This demonstrates a role for cutaneous immune cells in the inflammatory skin response to pollution, which may enable future hypothesis formation around the impact of pollution on skin and aid the development of novel topical treatments for pollution‐induced skin conditions. These models also offer an in vitro system that reproduces aspects of the immunological response of native skin, potentially reducing the need for animal models for pre‐clinical studies.

## 1 INTRODUCTION

The current environmental levels of air pollution worldwide pose a serious threat to global health^1^ and human skin constantly comes into contact with this stressor. The composition of air pollution is complex, consisting of several groups of compounds, including gaseous pollutants (such as NO_*x*_, CO, and ozone), particulate matter (PM), heavy metals, and polycyclic aromatic hydrocarbons (PAH).^2^, ^3^ PM is a widely studied component of air pollution due to its abundance in the urban atmosphere and its propensity to act as a carrier for other pollutants such as PAH and heavy metals through adsorption to the carbon core.^2^, ^4^ PM is generally categorized into three groups based on the particulates' diameter: PM_10_ (diameter 2.5–10 μm), PM_2.5_ (2.5–0.1 μm), and ultrafine PM (PM_0.1_, <0.1 μm).^2^, ^3^ Additionally, the chemical composition and surface structure of PM also vary, based on the source of the air pollution.^2^, ^5^ The size and composition of particles directly affect the health risks posed by the PM, and though the World Health Organization (WHO) currently only provides limits for the atmospheric concentration of PM_10_ and PM_2.5_, there is increasing evidence that the finer particles pose the greatest risk to human health.^2^, ^6^, ^7^

The damaging effects of PM on the respiratory and cardiovascular systems have long been established in the literature, with inhalation of particulate being linked to conditions ranging from tissue inflammation to heart failure and cancer.^8^ However, it is only in the last 15 years that the impact of PM on skin health has become a topic of research interest. Early epidemiological studies into the effect of pollution on skin demonstrated links between high environmental pollutants and increased signs of skin ageing^9^ and higher incidences of inflammatory skin conditions such as atopic dermatitis.^10^, ^11^ Most recently, research has utilized exposure of human skin equivalent (HSE) cultures to better understand the mechanisms that underpin these cutaneous changes. HSEs offer advantages over 2D cell cultures as they replicate the complex structure of native skin, including the formation of the epidermal barrier, which produces more in vivo‐like responses after topical exposure to environmental agents such as irritant compounds and ultraviolet radiation (UVR).^12^, ^13^, ^14^ Studies utilizing HSE cultures have demonstrated that exposure to air pollution induces inflammation with an increase in inflammatory cytokines including interleukin (IL)‐1α, IL‐6, and IL‐8.^15^, ^16^, ^17^, ^18^, ^19^, ^20^, ^21^ One study also demonstrated that particles could penetrate the stratum corneum and enter the deeper cell layers, suggesting a potential physical mechanism behind PM‐induced inflammation.^15^ However, most of this research utilized epidermal‐only HSE, and despite the suggestion of immunological involvement in the cutaneous response to PM, there have been no previous studies that have used immune‐competent full thickness (i.e., containing both an epidermal and dermal compartment) (FT‐)HSE to study these pathways.

Due to the role that cutaneous immune cells play in response to other exogenous stressors, such as UVR, many research groups have aimed to develop more complex HSE that more accurately reflect the skin's immune system. One method to recapitulate the *native* skin environment has been to integrate resident immune cells, such as Langerhans cells (LC), into HSE. LC are located in the epidermis, and act as sentinels that play important roles in maintaining homeostasis, and mediating inflammatory and adaptive immune responses.^22^, ^23^ The phenotype of primary isolated LCs can rapidly change in culture,^24^ and while LCs can be generated from peripheral blood monocyte cells (PMBC)^25^ and integrated into HSE cultures,^26^, ^27^, ^28^, ^29^, ^30^ their behavior differs between each individual blood donor.^24^ Due to this variability, cell lines, particularly the acute myeloid leukemic MUTZ‐3 lineage, have been used as a source of LC. These MUTZ‐3‐derived LC (MUTZ‐3‐LC) show similar structure and function to their in vivo counterparts while offering more reproducibility than patient‐derived monocytes.^24^, ^30^, ^31^, ^32^ Previously, MUTZ‐3‐LC have been successfully incorporated into FT‐HSE, and results of these studies have demonstrated not only that the MUTZ‐3‐LC in these models phenotypically resemble in vivo LC^33^ but also that they respond to topical allergen and irritant exposure by phenotype‐switching and migrating from the epidermis, as would occur naturally in native skin.^30^, ^34^, ^35^ Therefore, FT‐HSE‐containing LC may offer an in vitro platform to better study the skin's response to other external stressors, such as air pollution.

Here, we aim to assess the effect of PM exposure on the inflammatory response of skin through the utilization of an in vitro immuno‐competent FT‐HSE. Through adapting our previously established protocol,^36^ we have developed a FT‐HSE that contains MUTZ‐3‐LC, which we have characterized using immunofluorescence staining and cytokine analysis. We also demonstrate how our FT‐HSE models with MUTZ‐3‐LC show a greater response to allergen and irritant exposure than models without MUTZ‐3‐LC. We employ these equivalents as a platform to investigate the effect of PM on human skin. We used the standard reference material 1650b “diesel particulate matter” (DPM) from the National Institute of Standards and Technology (NIST, USA), which has an average particle diameter of 0.18 μm and contains various PAH, to model in vitro, the exposure of skin to urban air pollution. We have analyzed the physical interaction between DPM and the skin, using ultramicroscopic techniques to determine whether particulates had the ability to penetrate the skin. We have also measured the inflammatory cytokine output of PM‐exposed FT‐HSEs with or without MUTZ‐3‐LC to assess the ability of DPM to induce inflammation in the skin and the potential role of LC in this response.

## 2 RESULTS

### 2.1 Development of human skin equivalents containing MUTZ‐3‐derived Langerhans cells

**FIGURE 1:**
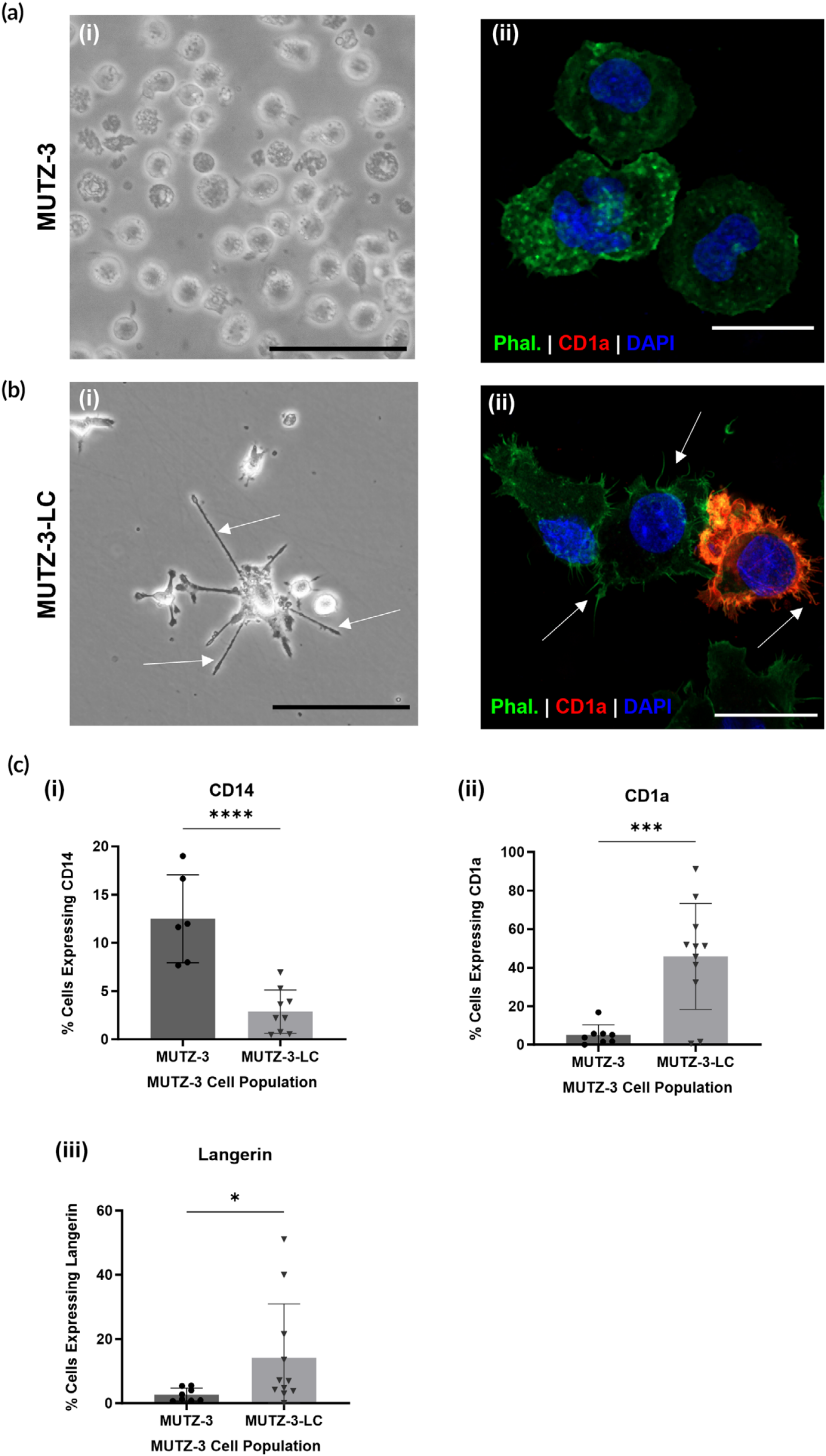
Differentiation of MUTZ‐3 cells into MUTZ‐3 Langerhans cells. (a) (i) Phase‐contrast image of MUTZ‐3 cells in suspension culture, (ii) immunocytochemistry image of MUTZ‐3 cells stained with phalloidin (Actin, green), anti‐CD1a (red), and DAPI (nuclei, blue). (b) (i) Phase‐contrast image of MUTZ‐3 differentiated into Langerhans cells (MUTZ‐3‐LC), (ii) immunocytochemistry image of MUTZ‐3‐LC stained with phalloidin (Actin, green), anti‐CD1a (red), and DAPI (nuclei, blue). Arrows indicate dendrites. (c) Flow cytometry analysis graphs displaying percentage of cells expressing (i) CD14, (ii) CD1a, (iii) Langerin, in MUTZ‐3 and MUTZ‐3‐derived Langerhans cells (MUTZ‐3‐LC) populations. Data represented as mean ± standard deviation (SD). Individual data points represent the combined replicates (*n* = 6–11) of two to three independent trials of the MUTZ‐3‐LC differentiation protocol described in Section 4. Statistical analysis via one‐tailed unpaired *t*‐test *p < 0.05, ***p < 0.001, ****p < 0.0001. Scale bars: (a, b) (i) 100 μm, (ii) 10 μm.

To generate LC for use in FT‐HSEs, we differentiated the human myelomonocytic cell line MUTZ‐3 using a cytokine cocktail as previously described by Kosten et al.^35^ MUTZ‐3 cells grow in suspension as spheroid blast‐like cells (Figure 1ai). After a 7‐day incubation with the cytokines granulocyte‐macrophage colony‐stimulating factor (GM‐CSF), tumor necrosis factor (TNF)‐α, and transforming growth factor (TGF)‐β, the cells show a dendritic morphology and adhere to the cell culture plastic (Figure 1bi). Fluorescent staining of the actin cytoskeleton also shows dendritic protrusions in the differentiated MUTZ‐3 cells (MUTZ‐3‐LC) (Figure 1bii). We also used immunocytochemistry to stain both undifferentiated and differentiated MUTZ‐3 cells for the dendritic cell marker CD1a and saw no staining in the undifferentiated cells but positive staining in the MUTZ‐3‐LC population (Figure 1aii, bii). To further assess the expression of Langerhans cell markers in the MUTZ‐3‐LC, we conducted flow cytometry analysis to compare the differentiated cells to the undifferentiated MUTZ‐3 population. The results of this analysis showed the differentiated MUTZ‐3 population expressed significantly less of the monocyte/macrophage marker CD14 and significantly more of the dendritic marker CD1a and the LC marker Langerin than the undifferentiated population (Figure 1c).

**FIGURE 2:**
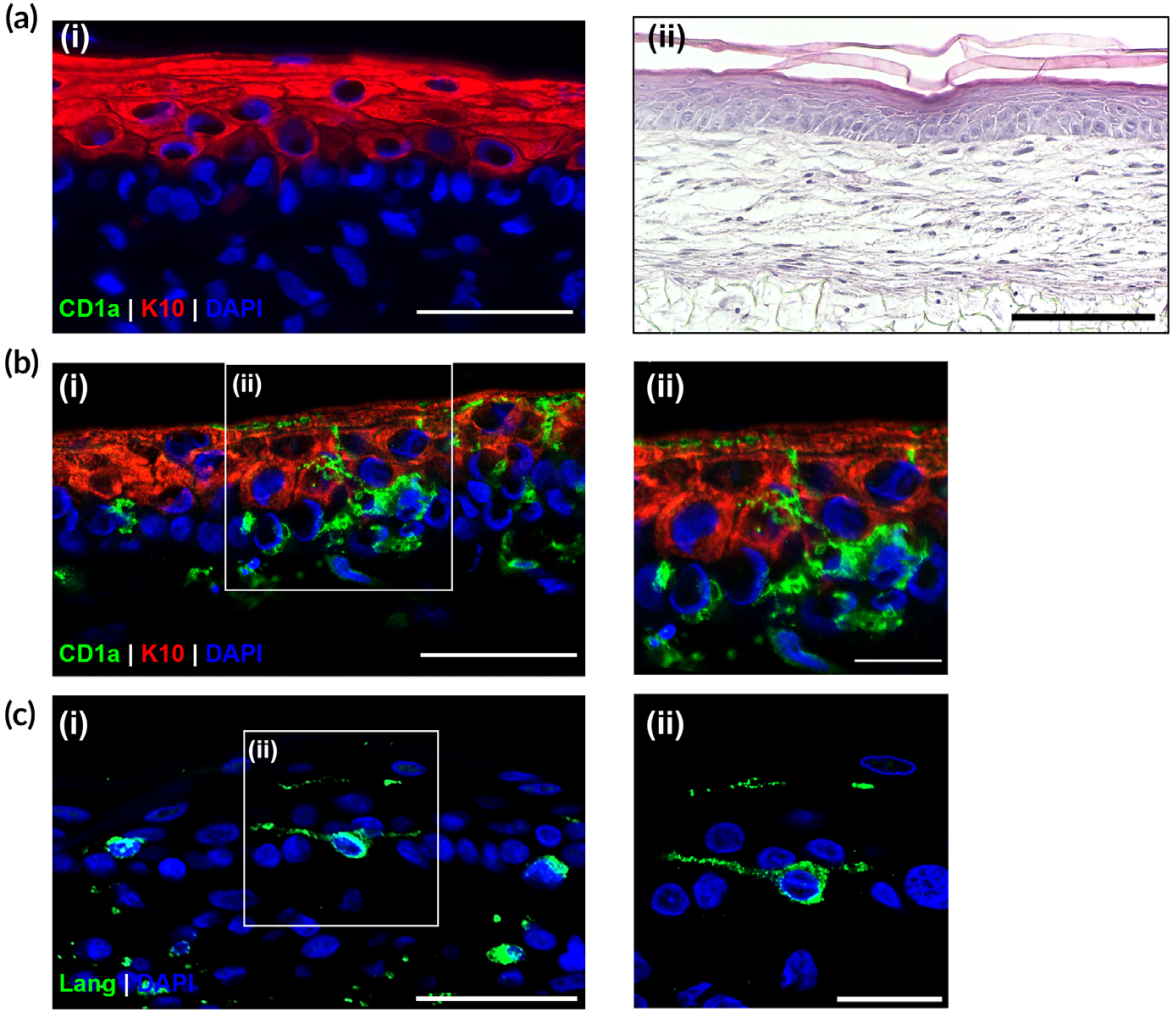
Incorporation of MUTZ‐3 differentiated into Langerhans cells (MUTZ‐3‐LC) into the epidermal compartment of a full‐thickness equivalent model. (a) (i) Immunofluorescent image of full‐thickness skin model stained for CD1a (green), cytokeratin 10 (K10, red), and DAPI (nuclei, blue), (ii) representative hematoxylin and eosin staining histology of a full‐thickness skin model. (b) (i) Representative immunofluorescent image of full‐thickness skin model generated with MUTZ‐3‐LC stained for CD1a (green), K10 (red), and DAPI (nuclei, blue), (ii) enlargement of boxed area of image (bi) that shows the dendrites of a MUTZ‐3‐LC between the keratinocyte layers. (c) (i) Representative immunofluorescent image of full‐thickness skin model generated with MUTZ‐3‐LC stained for Langerin (green), and DAPI (nuclei, blue), (c) (ii) higher magnification image of boxed area of image (ci). Scale bars: (a–c) (i) 50 μm, (a) (ii) 200 μm, (b–c) (ii) 20 μm.

Having generated a population of LC from the MUTZ‐3 cell line, we then integrated MUTZ‐3‐LC into FT‐HSE by adapting our previous protocol.^36^ Our FT‐HSE consists of a porous polystyrene scaffold containing human dermal fibroblasts, which produce endogenous extracellular matrix, forming a dermal compartment that supports a stratified and cornified epidermis generated from human epidermal keratinocytes (Figure 2a). FT‐HSE without MUTZ‐3‐LC does not contain any dendritic cells or LC, as demonstrated by the negative staining for CD1a (Figure 2ai). We generated FT‐HSE with MUTZ‐3‐LC by co‐seeding the keratinocytes with the MUTZ‐3‐LC onto the dermal compartment. Immunofluorescent analysis of these models reveals CD1a‐positive and Langerin‐positive MUTZ‐3‐LC present in the epidermis (with some cells present in the upper fibroblast layers) (Figure 2b,c). Overall, MUTZ‐3‐LC are primarily located in the basal and suprabasal layers of the epidermis and show a dendritic morphology, with dendrites that extend between the surrounding keratinocytes in the viable layers (Figure 2bii).

These staining data show that we have successfully generated a FT‐HSE containing CD1a+/Langerin+ LC with dendritic morphology that are primarily localized to the layers of the epidermis, which is comparable to native human skin.

### 2.2 Skin equivalent models containing MUTZ‐3‐LCs show a distinct cytokine profile

LC in the epidermis function as sentinels that sample their environment for foreign substances, as antigen‐presenting cells that migrate to the lymph node to initiate T‐cell responses, and also act to remove post‐apoptotic keratinocytes.^37^ To examine how the MUTZ‐3‐LC function in our FT‐HSE models, we compared the secreted cytokine levels of these models to FT‐HSE without MUTZ‐3‐LC using the Eve Technologies Discovery Assay® cytokine array.

**FIGURE 3:**
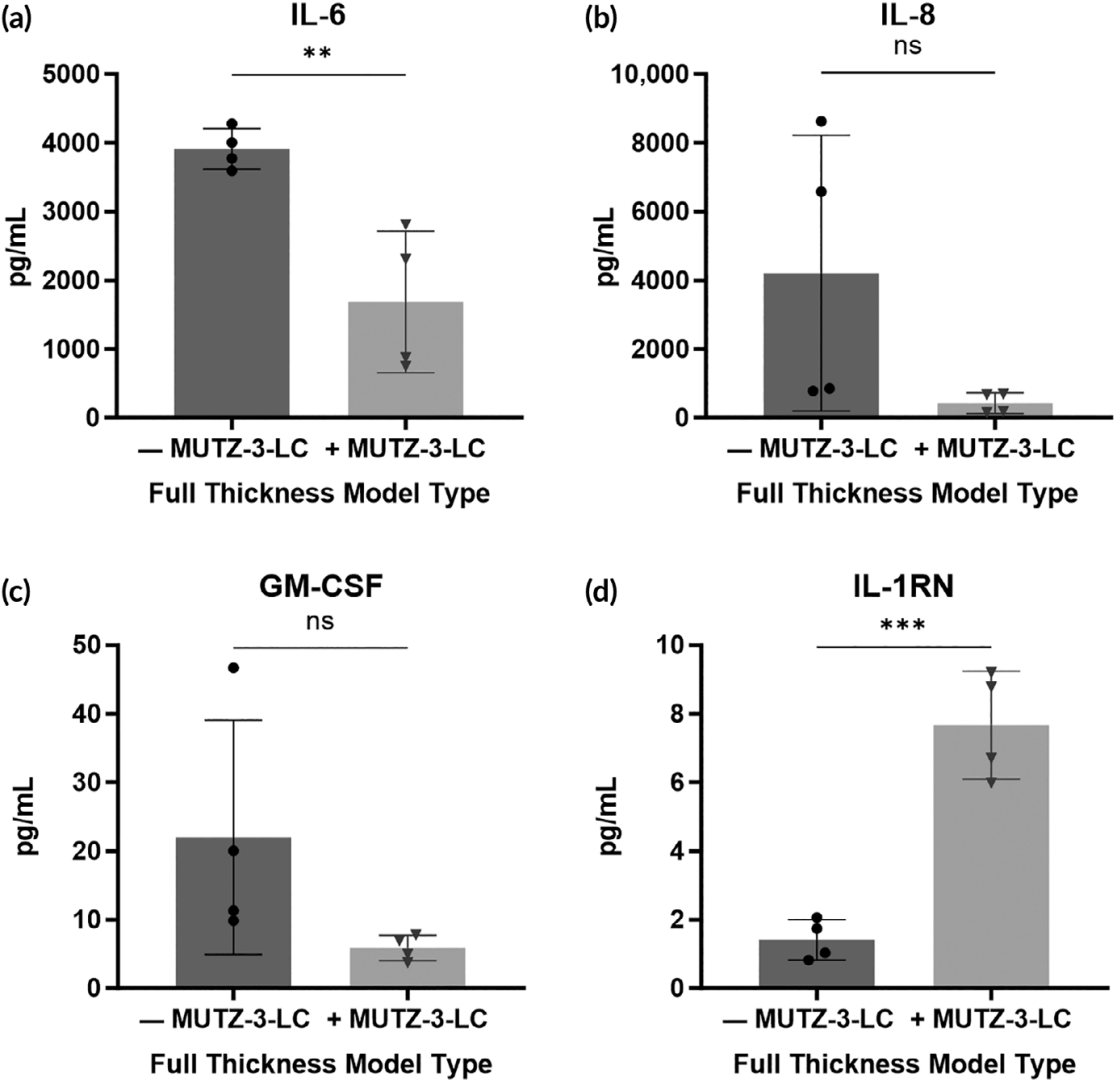
Cytokine expression levels in full‐thickness skin equivalents with and without MUTZ‐3 differentiated into Langerhans cells (MUTZ‐3‐LC). Graphs showing cytokine concentration of (a) interleukin (IL)‐6, (b) IL‐8, (c) granulocyte‐macrophage colony‐stimulating factor (GM‐CSF), (d) IL‐1 receptor antagonist (IL‐1RN) in the media of full‐thickness models with and without MUTZ‐3‐LC. Data represented as mean concentration ± SD. Individual data points represent the combined replicates (*n* = 4) of two independent human skin equivalents generation experiments as described in Section 4. Statistical analysis via two‐tailed unpaired *t*‐test, *n* = 4. **p < 0.01, ***p < 0.001.

Quantification of the levels of proinflammatory cytokine IL‐6 showed that the protein level was significantly lower in the MUTZ‐3‐LC‐containing model compared to the standard FT‐HSE model (Figure 3a). Additionally, levels of the inflammatory cytokines IL‐8, and GM‐CSF were also lower in the FT‐HSEs with MUTZ‐3‐LCs, though not significantly (Figure 3b,c). These results suggest that FT‐HSE models with MUTZ‐3‐LCs have a lower proinflammatory cytokine profile than FT‐HSE without immune cells. We also analyzed levels of IL‐1 receptor antagonist (IL1‐RN), which can have an anti‐inflammatory function in skin, and showed that the protein levels were significantly higher in the equivalents with MUTZ‐3‐LCs (Figure 3d). We did observe some inter‐experimental variations in cytokine levels (e.g., Figure 3b—MUTZ‐3‐LC) which could be attributed to different keratinocytes and fibroblasts or to other factors that differ between HSE set ups. Overall, however, these data suggest that under normal culture conditions, FT‐HSE with MUTZ‐3‐LCs have a lower proinflammatory and higher anti‐inflammatory cytokine levels compared to skin equivalents without immune cells.

To characterize the function of MUTZ‐3‐LC in mediating inflammatory responses in FT‐HSE, we treated models with compounds that produce an allergic (10 mM nickel sulfate, NiSO_4_) or irritant (2.5 mg/mL sodium dodecyl sulfate [SDS]) immune response. We assessed inflammation by measuring the media cytokine levels produced after treatment in the FT‐HSE with or without MUTZ‐3‐LCs. As the baseline levels of cytokines in the two groups of models differed significantly (Figure 3) we analyzed the data by calculating the fold change of the cytokine as compared to the untreated control of the same FT‐HSE type.

**FIGURE 4:**
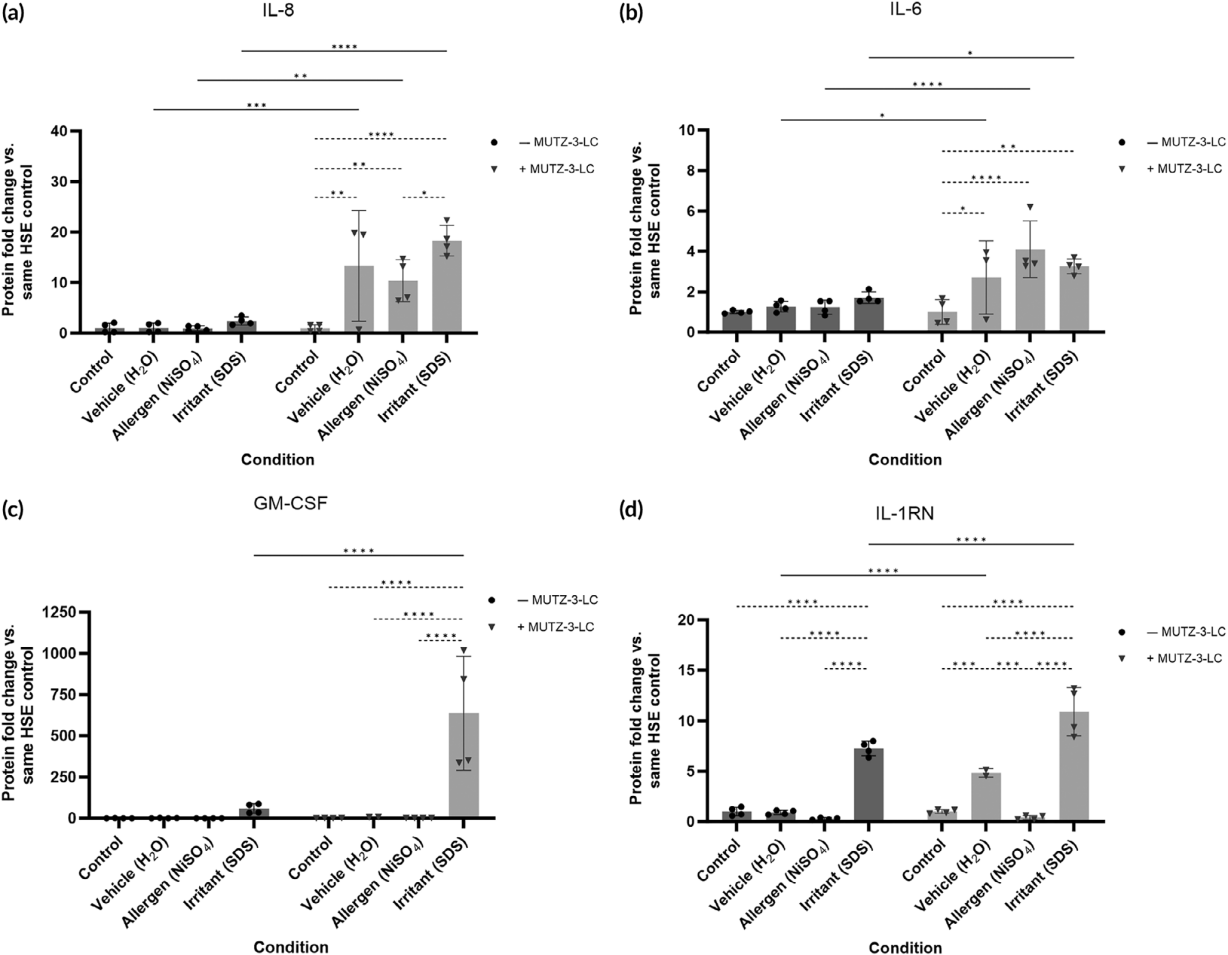
Changes to cytokine expression after topical treatment with an allergen or irritant in full‐thickness skin equivalents with and without MUTZ‐3 differentiated into Langerhans cells (MUTZ‐3‐LC). Graphs showing media cytokine levels of (a) interleukin (IL)‐8, (b) IL‐6 (c) granulocyte‐macrophage colony‐stimulating factor (GM‐CSF), (d) IL‐1 receptor antagonist (IL‐1RN) in full‐thickness models with and without MUTZ‐3‐LC exposed to vehicle (H_2_O), allergen (NiSO_4_), or irritant (sodium dodecyl sulfate). Data represented as mean fold change versus same‐type human skin equivalents (HSE) control ± SD. Individual data points represent the combined replicates (*n* = 2–4) of two independent HSE generation experiments as described in the methods section. Statistical analysis via two‐way ANOVA (Analysis of Variance) with Tukey's test for multiple comparisons. *p < 0.05, **p < 0.01, ***p < 0.001, ****p < 0.0001.

In FT‐HSEs lacking MUTZ‐3‐LCs, there was no significant fold increase in the proinflammatory cytokines IL‐8 and IL‐6 in any treatment condition, whereas, the vehicle, allergen, and irritant‐treated MUTZ‐3‐LC FT‐HSE models showed a significant increase in IL‐8 and IL‐6 production compared to the MUTZ‐3‐LC control (Figure 4a,b). Additionally, FT‐HSE with MUTZ‐3‐LC treated with irritant showed a large, significant increase in GM‐CSF expression, but, as with IL‐8 and IL‐6, there was no significant response in the equivalent without MUTZ‐3‐LCs (Figure 4c). Our analysis also showed that the expression of the anti‐inflammatory mediator IL‐1RN increased significantly after irritant treatment in FT‐HSE with and without MUTZ‐3‐LC, but the increase was significantly larger in the MUTZ‐3‐LC‐containing equivalents (Figure 4d). Although, FT‐HSE with MUTZ‐3‐LC also showed a significant increase in IL‐1RN in response to the vehicle, when compared to the irritant condition, the increase was significantly lower (Figure 4d). Overall, these data suggest that FT‐HSEs without an immune component show a lower proinflammatory and anti‐inflammatory response to topical irritant exposure when compared to equivalents with MUTZ‐3‐LCs. Additionally, only models with MUTZ‐3 showed a significant increase in IL‐8 and IL‐6 after treatment with allergen, and only these models responded to H_2_O exposure.

### 2.3 Diesel particulate matter does not penetrate the stratum corneum of skin equivalents

**FIGURE 5:**
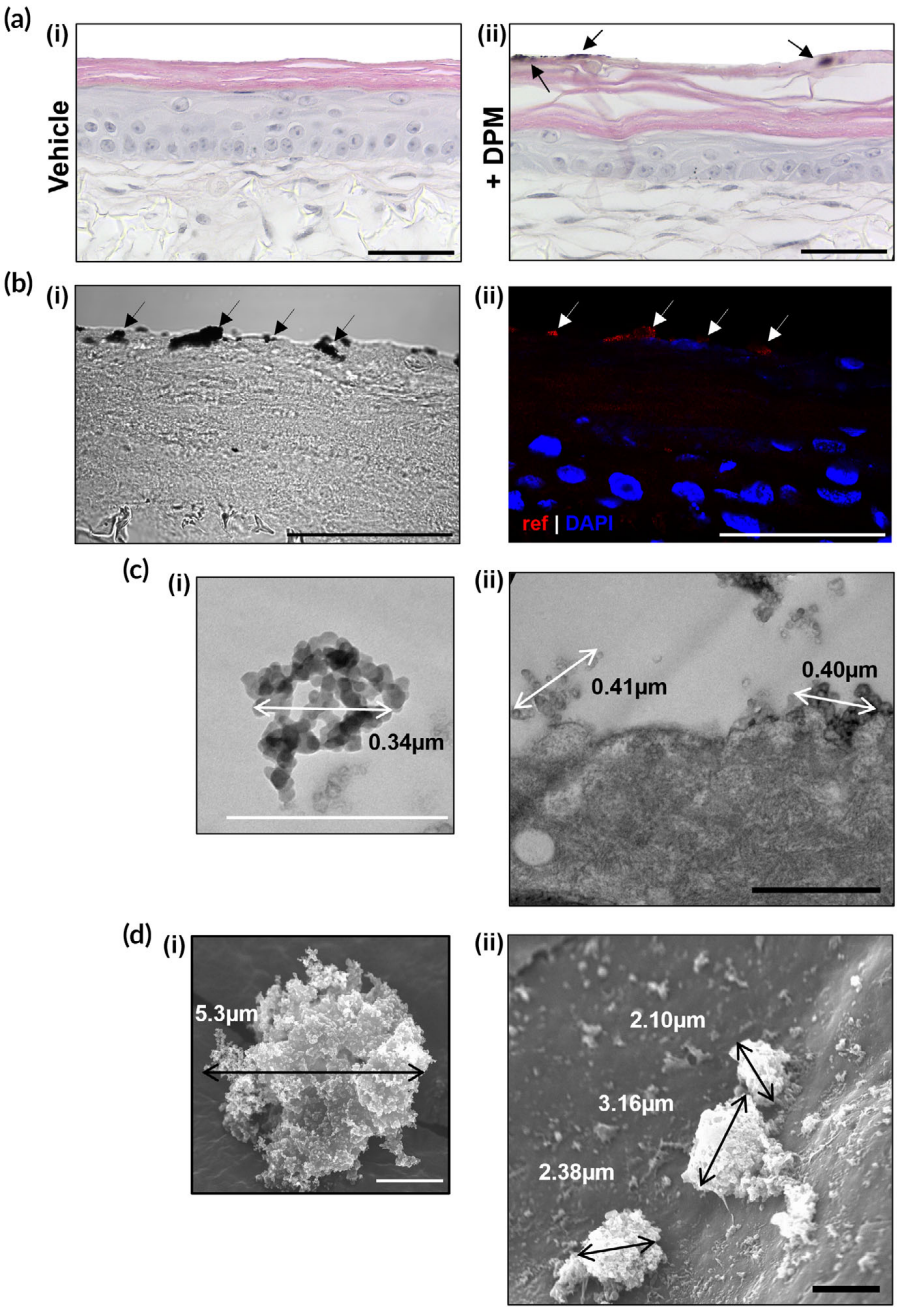
Histological, fluorescent and electron microscopic assessment of skin equivalents treated with diesel particulate matter. (a) Hematoxylin and eosin staining of (i) vehicle and (ii) diesel particulate matter (DPM) treated full‐thickness skin equivalents. Arrows indicate visible particulate matter. (b) Full thickness skin model treated with DPM labeled with DAPI (nuclei, blue) imaged with (i) brightfield and (ii) light microscopy. Black arrows indicate visible DPM, white arrows indicate reflectance (red) of particulate. (c) Transmission electron microscopy of (i) DPM and (ii) uppermost stratum corneum layer of a skin equivalent treated with DPM. Measurements indicate the width of the particulates. (d) Scanning electron microscopy of (i) DPM and (ii) the surface of a skin equivalent treated with DPM. Measurements indicate the width of the particulates. Scale bars: (a, b) 50 μm, (c) 0.5 μm, (d) 2 μm.

To investigate the effect of topical exposure to DPM on the FT‐HSE models, we first assessed whether the particles could penetrate past the stratum corneum and reach the viable layers of keratinocytes. Hematoxylin and eosin staining (H&E) staining of vehicle (liquid paraffin) and DPM‐exposed skin equivalents demonstrated that particles adhere to the surface of the skin (Figure 5a). However, deeper penetration of particles could not be clearly assessed using this stain, so we investigated whether the physical properties of DPM would allow the location of particles to be imaged using fluorescent light microscopy. We discovered that though the particles were not fluorescent, they were reflective, and by using an Ex/Em spectrum of 405 nm/405 nm, they could be imaged without staining (Figure 5bii). We then compared brightfield and fluorescent images of the same section and utilized the reflective nature of the PM to assess if any black particles that appeared on the surface and deeper in the skin were DPM (Figure 5b). Light microscopy did not reveal any deeper particle penetration, but the reflective properties of epidermal components (such as granules in the granular layer) make it difficult to conclude this for certain; therefore, we employed electron microscopy techniques to view the interaction between the particles and skin at a higher resolution.

First, we imaged DPM using transmission (TEM) and scanning (SEM) electron microscopy (EM) to visualize the ultrastructure of the particulates in isolation. TEM images of DPM showed that the particles consist of aggregates approximately 0.5 μm in diameter made up of smaller individual particles (Figure 5ci). This aciniform organization, also seen in the SEM images, leads to the particles having an irregular structure with a large surface area, which may contribute to the adhesive abilities of DPM (Figure 5di). We then imaged skin equivalents exposed to DPM using TEM and SEM to investigate if these particulate structures could be seen within the layers of the epidermis. TEM images of ultrathin stained sections of DPM‐exposed skin models showed irregular particulate aggregates adhered to the uppermost corneocyte of the epidermis (see Figure S1a) but did not show any evidence of DPM deeper in the skin (Figure 5cii). We also analyzed the surface of DPM‐exposed skin equivalents using SEM. These images showed DPM aggregates in a range of sizes on the surface of the skin; however, as with the TEM data, there was no evidence of particles between the corneocytes, which further suggested that DPM does not penetrate deeper than the uppermost stratum corneum (Figure 5dii).

Through various imaging techniques, we determined that the DPM could not penetrate deeper than the uppermost stratum corneum of our skin equivalents. However, even without reaching the viable layer, DPM may still trigger inflammatory responses in the skin via other mechanisms, for example, the transport of adhered chemicals, such as PAH, through the lipophilic stratum corneum or by oxidizing these lipids, generating increased oxidative stress in deeper cell layers.

### 2.4 Full thickness skin equivalents containing MUTZ‐3‐LCs demonstrate a proinflammatory response to diesel particulate matter exposure

Previously we demonstrated that FT‐HSE containing MUTZ‐3‐LCs showed increased inflammatory cytokine release after exposure to known skin stressors (NiSO_4_ and SDS) compared to skin equivalents without immune cells (Figure 4). We then aimed to assess two hypotheses: first, that DPM exposure increases inflammation in skin equivalents and second, that FT‐HSEs containing MUTZ‐3‐LCs would show a heightened cytokine response when exposed to these particulates.

**FIGURE 6:**
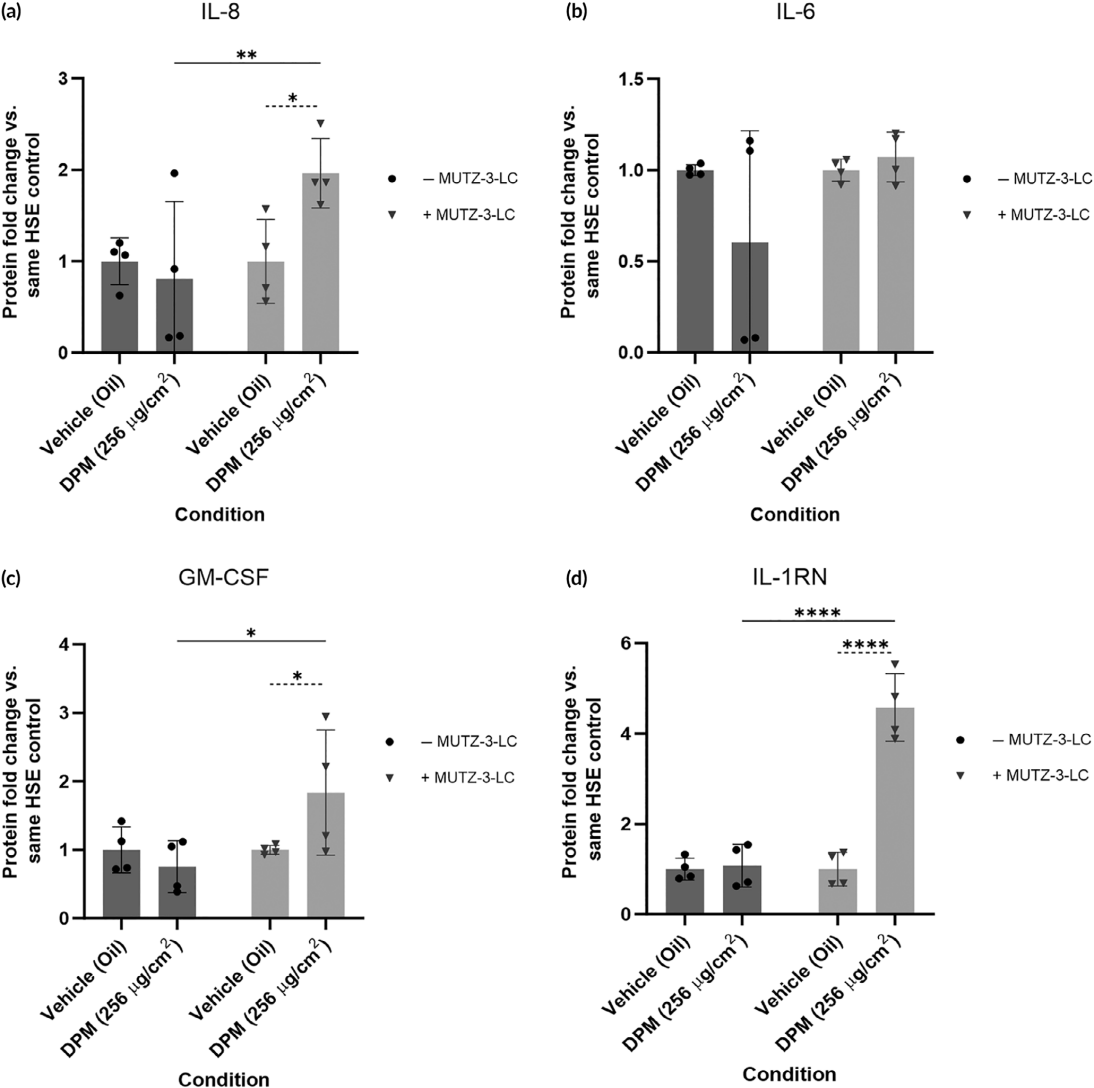
Cytokine expression changes in full‐thickness models with and without MUTZ‐3 differentiated into Langerhans cells (MUTZ‐3‐LC) after treatment with diesel particulate matter. Graphs showing the media cytokine levels of (a) interleukin (IL)‐8, (b) IL‐6, (c) granulocyte‐macrophage colony‐stimulating factor (GM‐CSF), (d) IL‐1 receptor antagonist (IL‐1RN) in full‐thickness models with and without MUTZ‐3‐LC exposed to 256 μg/cm^2^ of diesel particulate matter (DPM). Data represented as mean fold change versus same‐type human skin equivalents (HSE) vehicle (oil) control ± SD. Individual data points represent the combined replicates (*n* = 4) of two independent HSE generation experiments as described in the methods section. Statistical analysis via two‐way ANOVA with multiple comparisons. *p < 0.05, **p < 0.01, ****p < 0.0001.

To evaluate this, we exposed FT‐HSE topically to either the vehicle (liquid paraffin oil) or 256 μg/cm^2^ DPM for 1 week as a proxy for chronic, high‐level pollution exposure. Media was harvested after the final 24 h of the exposure and analyzed using the Eve Technologies Discovery Assay® cytokine array. We assessed the data by comparing the protein fold change of the DPM‐exposed models versus the vehicle control of the same FT‐HSE type. The FT‐HSE without MUTZ‐3‐LCs showed no significant fold increase in the levels of the proinflammatory cytokines IL‐8, IL‐6, or GM‐CSF (Figure 6a–c). In contrast, there was a significant increase in the production of IL‐8 and GM‐CSF in MUTZ‐3‐LC‐containing models, which was not seen in the models without MUTZ‐3‐LCs (Figure 6a,c). Analysis of the levels of IL‐1RN also showed that there was no significant change in IL‐1RN production in FT‐HSEs without MUTZ‐3‐LCs but that there was a significant 4.58‐fold increase in the equivalents containing immune cells (Figure 6d).

We previously showed that equivalents without MUTZ‐3‐LC have a lower cytokine response to known inducers of inflammation (NiSO_4_ and SDS) (Figure 4). The results of this analysis further demonstrate this decreased response of FT‐HSE to stressors, as 256 μg/cm^2^ DPM did induce an increase in inflammatory cytokines, but only in the FT‐HSEs that contain MUTZ‐3‐LCs (Figure 6).

## 3 DISCUSSION

Legislation that limits the use of animal testing for the assessment of cosmetics has driven a need to develop FT‐HSEs that better reflect the in vivo responses of the skin.^38^, ^39^ The cutaneous immune environment plays a significant role in the inflammatory response to chemical (e.g., allergens and irritant compounds) and environmental stressors (e.g., UVR). Accordingly, the inclusion of functional immune cells such as LC into FT‐HSEs has become a vital step in the move toward more accurate in vitro testing. Here we have described the development of a FT‐HSE that contains epidermally situated MUTZ‐3‐derived LCs that closely resemble the structural and phenotypic hallmarks of LCs in native skin. We have also characterized the functionality of this FT‐HSE MUTZ‐3‐LC model by demonstrating recapitulation of the in vivo inflammatory response to topical allergen and irritant exposure. Furthermore, we have built on this work by using this immune‐competent skin platform to demonstrate the involvement of LC in PM‐induced inflammation.

Various research groups have previously reported the ability to generate a LC population from either primary peripheral blood mononuclear cells or from the MUTZ‐3 cell line. Due to issues in donor variation and availability of patient‐derived monocytes, we selected to use commercially available MUTZ‐3 cells as the progenitor pool for LC generation. We utilized previously described protocols for MUTZ‐3‐LC generation^32^, ^35^ and as previously reported, were able to successfully differentiate the MUTZ‐3 cell line into a LC population. Our MUTZ‐3‐LC population showed the characteristic dendritic morphology of isolated primary LCs, and we also demonstrated the positive expression of the dendritic cell marker CD1a and the LC marker Langerin. We then generated immune‐competent FT‐HSE by adapting our previous protocol and integrating MUTZ‐3‐LCs into the epidermal compartment. Our results show CD1a+ and Langerin+ MUTZ‐3‐LC situated in the epidermis of the FT‐HSE with dendrites extended between the keratinocytes of the epidermal strata, reflecting in vivo tissue structure.^40^ Therefore, in contrast to previous HSEs containing MUTZ‐3‐LC,^30^, ^33^, ^35^ we have produced a fully human immune‐competent FT‐HSE containing no animal‐derived or exogenous collagens, made entirely from commercially available primary human keratinocytes and fibroblast and from human MUTZ‐3 cell line‐derived LCs.

Under normal conditions, epidermal LCs play a role in maintaining cutaneous homeostasis by regulating levels of inflammation in the tissue.^22^, ^23^ Through analysis of the cytokine production in FT‐HSEs with and without MUTZ‐3‐LCs, we demonstrated that models containing LCs had lower basal levels of proinflammatory cytokines IL‐8, IL‐6, and GM‐CSF (with IL‐6 being significantly lower) and significantly higher amounts of the anti‐inflammatory IL1‐RN. These data agree with previous results that showed lower levels of IL‐8 and IL‐6 in HSE‐containing monocyte‐ and MUTZ‐3‐derived LCs compared to models without LCs.^30^ This therefore suggests that the MUTZ‐3‐LCs in our FT‐HSEs act as regulators of cytokine release, maintaining a non‐inflammatory environment, which mimics the functions of resident immune cells in real tissue.^22^, ^41^

In this paper, we also demonstrate the immunological function of our MUTZ‐3‐LC FT‐HSE in response to topical application of known cutaneous stressors. In human skin, contact allergenic compounds, such as NiSO_4_, can penetrate the epidermal barrier, and these haptens trigger immune responses in keratinocytes and LC, leading to an increase in proinflammatory cytokines such as IL‐1 and IL‐8.^42^, ^43^ Irritants, such as SDS, induce an immune response by directly damaging the structure of skin cells, leading to an increase in cytokine production from keratinocytes and LC, including IL‐6, IL‐8, GM‐CSF, and IL‐1RN.^44^, ^45^, ^46^ The allergenic response of skin to sensitizers has been studied using in vitro HSEs that include LC, with some research finding LC are required for contact allergen‐induced increase in cytokines, such as IL‐8 and IL‐6,^30^, ^47^ whereas others demonstrated an increase in the absence of immune cells.^48^ We found that NiSO_4_ induced a significant increase in the fold change of IL‐8 and IL‐6 but only in FT‐HSEs with MUTZ‐3‐LCs, demonstrating that the MUTZ‐3‐LC may replicate the response of LCs to allergens in vivo.^49^ In turn, this may imply that HSEs without LC may fail to replicate such a response. In line with some previous research, we saw a significant increase in proinflammatory cytokine levels after exposure to the irritant SDS in models with MUTZ‐3‐LCs, but not in FT‐HSE without.^30^ Additionally, we found that while IL‐1RN levels increase in both FT‐HSE treated with SDS, the response was greater in FT‐HSEs containing immune cells. Together these results demonstrate in vitro the important role of LCs in cutaneous irritant‐induced inflammation. Another major function for LCs in the cutaneous response to both allergens and irritants is the activation of T‐cell responses via migrating from the skin to the lymph node.^42^, ^44^ Previous FT‐HSEs that have included MUTZ‐3‐LC have demonstrated this functionality in their models.^30^, ^34^, ^35^, ^50^, ^51^ Thus, this would be the obvious next step in further characterizing our immune‐competent FT‐HSE. Additionally, the keratinocytes and fibroblasts used to generate these models were sourced from male, light‐moderately pigmented, neonatal donors. Sex, ethnicity, and age are demographic factors that would contribute to different skin morphology and function.^52^, ^53^, ^54^ Therefore, an interesting line of future research would be to generate skin equivalents from different donor backgrounds (i.e., different age categories) to compare the inflammatory responses.

Having demonstrated the ability of our models to recapitulate some of the hallmark cytokine responses produced in real skin after allergen and irritant treatment, we then aimed to use the platform to assess the cutaneous immunological reaction to air pollution exposure. Previous studies that have utilized epidermal HSEs to investigate the impact of PM (of various sizes and compositions) or PM‐containing substances (such as tobacco smoke) on skin have consistently demonstrated that pollution induces inflammation, including increased expression of proinflammatory cytokines such as IL‐1α,^15^, ^16^, ^17^, ^19^, ^21^ IL‐6,^19^ and IL‐8.^17^, ^18^, ^19^, ^21^ This corroborates findings from PM‐exposed 2D keratinocyte cultures^11^, ^55^, ^56^ and evidence from epidemiological studies that exposure to air pollution can trigger or worsen cutaneous inflammation‐associated disorders.^11^ Additionally, HSE containing LCs have been shown to recapitulate some of the in vivo cutaneous immunological responses triggered by solar UVR exposure, another environmental agent, including a reduction in the number of LCs in the epidermis, LC morphological changes such as loss of dendricity and changes in LC cell marker expression.^29^, ^57^, ^58^ Despite the established involvement of LCs in other types of stress‐induced cutaneous inflammation, and evidence of pollution‐induced increases in proinflammatory cytokines, this is the first time, to our knowledge, that a FT‐HSE containing LCs has been used to study the immunological responses of the skin to air pollution. Reviewing the results from these papers and from the data on the response of our MUTZ‐3‐LC FT‐HSEs to other topical stressors, we hypothesized that equivalents containing immune cells would show higher cytokine production in response to PM exposure. Our results showed that 1 week exposure to 256 μg/cm^2^ DPM induced no significant change in the production of cytokines in standard FT‐HSE; however, in models containing MUTZ‐3‐LCs, there was a significant increase in proinflammatory IL‐8 and GM‐CSF production and a significant increase in secretion of the regulatory molecule IL‐1RN. Previous evidence clearly shows LC involvement in the regulation of inflammation in the skin after treatment with exogenous stressors such as allergens, irritants, and UVR.^30^, ^35^, ^58^ Our data builds on this by suggesting that LCs play a role in the cutaneous inflammatory response to PM exposure, a component of environmental pollution, which suggests a requirement for the inclusion of immune cells in HSE cultures when aiming to investigate air pollution‐induced inflammation. More in‐depth investigation into the specific function of LCs in pollution‐driven cutaneous inflammation is required, since our research does not identify the mechanisms by which LC are activated, nor whether the increased cytokine levels are due to production by the LCs themselves, or by cellular signals from the LCs inducing increased cytokine production in the surrounding keratinocytes, or both. Additionally, we did not assess morphological changes of the MUTZ‐3‐LC within the HSE after topical stressor exposure or evaluate migration of LC from the epidermis, which is another area requiring further study.

To investigate the physical interaction between topically applied DPM and the epidermis, we used various microscopy techniques to image the structure of treated skin. One study that utilized epidermal HSEs suggested that PM could cause cutaneous damage by penetrating the stratum corneum and traveling deeper into the epidermis.^15^ However, there is conflicting evidence in the literature about whether particulates can penetrate through the stratum corneum, as a study using a murine in vivo model, and a different paper that utilized ex vivo human skin found that PM could not enter deeper than the superficial stratum corneum layers.^59^, ^60^ Therefore, we utilized various techniques to assess this in our epidermal HSEs treated with DPM. PM has a large specific surface area and chemical composition that makes it highly adherent to biological surfaces, such as the stratum corneum of skin.^61^ This was demonstrated herein using light microscopy, TEM, and SEM imaging that showed evidence of particulates attached to the surface of the skin model. However, analysis of our HSEs using both EM techniques did not show any evidence of DPM penetration past the uppermost stratum corneum, agreeing with evidence from Lee et al.,^60^ demonstrating the barrier function of the skin is sufficient to prevent particulate entry. These data also suggest that cutaneous damage by PM is not the trigger for the inflammatory response seen after topical DPM exposure and therefore another mechanism, such as diffusion of PAH through the stratum corneum^62^ or oxidative stress generation,^15^, ^21^ may be the cause.

In this study we have presented the development and characterization of a novel immune‐competent FT‐HSE model generated without the use of exogenous collagen matrixes utilizing commercially available primary keratinocytes and fibroblasts and MUTZ‐3 cell line‐derived LCs. We describe the correct localization of MUTZ‐3‐LCs in the epidermal compartment, where these cells show the typical dendritic morphology and CD marker expression of their in vivo counterparts. Furthermore, we demonstrated that FT‐HSEs with MUTZ‐3‐LC have lower basal levels of proinflammatory cytokines but show increased cytokine production after topical allergen and irritant exposure compared to models without immune cells, thus better recapitulating the responses of real skin. To build on this, we then utilized this immune‐competent FT‐HSE platform to investigate the response of skin to DPM exposure and found diesel particulates induced an increase in inflammatory cytokine production in models containing MUTZ‐3‐LCs. This suggests a potential role of LC responses in air pollution‐induced cutaneous inflammation.

Overall, the findings of this study show that our human FT‐HSE with MUTZ‐3‐LCs recapitulates the structure and inflammatory function of native skin, and we have also demonstrated how this model can be used to study the immunological impact of an important environmental stressor, air pollution.

## 4 MATERIALS AND METHODS

### 4.1 Cell culture

#### 4.1.1 Generation of MUTZ‐3‐derived Langerhans cells

MUTZ‐3 cells (male, acute myelomonocytic leukemia) (Deutsche Sammlung von Mikroorganismen und Zellkulturen [DSMZ], Braunschweig, Germany) were cultured in Minimum Essentials Medium Alpha (MEM α, Gibco) supplemented with 20% heat‐inactivated fetal bovine serum, 2 mM L‐Glutamine, 1 mg/mL penicillin–streptomycin, and 20% conditioned medium from the 5637 cell line (DSMZ). MUTZ‐3 cells were differentiated into LCs (MUTZ‐3‐LCs) by culturing at 1 × 10^5^ in growth media with the addition of 100 ng/mL GM‐CSF, 2.5 ng/mL TNF‐α, 50 μM 2‐mercaptoethanol, and 10 ng/mL TGF‐β (ThermoFisher Scientific, Loughborough, UK) for 7 days. Cells were cultured at 37°C, 5% carbon dioxide, and 95% humidity.

#### 4.1.2 Generation of skin equivalents

HSEs were generated as previously described^36^ using a 24‐well Alvetex® scaffold. Human epidermal neonatal keratinocytes (HEKn, male, light to moderate pigmentation) lot numbers #1817888, #1803415, #1944927, #2018512, #2286109, and human neonatal dermal fibroblasts (HDFn, male) lot numbers #1366356 and #1366434 (ThermoFisher Scientific) were used to produce HSE. To generate FT‐HSEs containing MUTZ‐3‐LCs, HEKn and MUTZ‐3‐LCs were seeded at a ratio of 1:1 onto 28‐day dermal compartments. Cultures were maintained for 48 h in submerged culture before moving to the ALI for a further 10 days of differentiation.

#### 4.1.3 Topical treatment of HSE

A 10 mM solution of nickel (II) sulfate hexahydrate (Sigma Aldrich, Dorset, UK) and a 2.5 mg/mL solution of SDS (Sigma Aldrich) were used as allergen and irritant solutions, respectively. DPM NIST® SRM® 1650b (Sigma Aldrich) was suspended in liquid paraffin (Johnson's® Baby Oil) at a stock concentration of 76,800 μg/mL and diluted 1:4 to create a 256 μg/cm^2^ solution. Ten microliters of allergen, irritant, DPM or respective vehicle solutions were added to the apical surface of HSE and spread evenly with a glass rod. HSE were incubated for 1 week before harvesting for downstream analysis.

### 4.2 Flow cytometry

Cell staining was performed using rabbit anti‐human CD14 (ab183322, Abcam, Cambridge, UK), mouse anti‐human CD1a (ab201337, Abcam), mouse anti‐human langerin (MA5‐24078, Invitrogen, ThermoFisher Scientific) primary antibodies and donkey anti‐rabbit IgG Alexa Fluor® 488, donkey anti‐mouse IgG Alexa Fluor® 488 (ThermoFisher Scientific) secondary antibodies. Isotype controls were used to assess non‐specific staining. Cells were incubated in primary and secondary antibody in phosphate‐buffered saline (PBS) containing 0.1% bovine serum albumin (BSA) for 1 h on ice and resuspended in the same buffer for flow cytometry analysis on a Guava EasyCyte flow cytometer.

### 4.3 Histology and hematoxylin and eosin staining

HSEs were fixed in 4% paraformaldehyde, dehydrated through a series of ethanol, and embedded in paraffin wax as described in Roger et al.^36^ Wax blocks were sectioned using a microtome (Leica RM2125RT, Leica, Milton Keynes, UK), and 5 μm sections were placed onto charged microscope slides (ThermoFisher Scientific). H&E was with Mayer's H&E (Sigma Aldrich) as described in Roger et al.^36^

### 4.4 Immunofluorescence staining

Sterile coverslips were coated with 10 μg/mL of poly‐D‐lysine (Sigma‐Aldrich) solution. MUTZ‐3 and MUTZ‐3‐LCs were seeded onto coverslips and incubated for 20 min at 37°C. Coverslips were fixed in 4% formaldehyde overnight at 4°C. Coverslips were then blocked and permeabilized in 20% neonatal calf serum (NCS, Sigma Aldrich) in 0.4% Triton‐X‐100 PBS for 45 min at room temperature and incubated in primary antibody (anti‐mouse CD1a) for 1 h at room temperature. Coverslips were washed three times in PBS and incubated in secondary antibody (donkey anti‐mouse IgG Alexa Fluor® 594) and Acti‐stain™ 488 Fluorescent Phalloidin (Cytoskeleton, Inc., Denver, CO, USA) for 1 h at room temperature. Coverslips were mounted in hard‐set Vectashield with DAPI (4′,6‐diamidino‐2‐phenylindole) (Vector Laboratories, Peterborough, UK), and slides were imaged on Zeiss 800 microscope.

Wax sections were deparaffinized in Histoclear (Scientific Laboratory Supplies, Nottingham, UK) and rehydrated through a series of ethanol. Antigen retrieval was achieved by incubating slides in citrate buffer for 20 min in a 95°C water bath followed by blocking and permeabilization with 20% NCS/0.4% Triton‐X‐100 for 1 h. Sections were incubated overnight at 4°C in primary antibodies: rabbit anti‐human cytokeratin 10 (ab111447, Abcam), mouse anti‐CD1a, mouse anti‐human CD207 (Langerin) (67788‐1‐Ig, Proteintech, Manchester, UK), and incubated for 1 h at room temperature in secondary antibody: donkey anti‐mouse IgG Alexa Fluor® 488 or donkey anti‐rabbit IgG Alexa Fluor® 594, plus Hoechst 33342 (ThermoFisher Scientific). Slides were mounted in soft‐set Vectashield (Vector Laboratories) and sealed with nail varnish. Slides were imaged on either a Zeiss 800 or Zeiss 880 confocal microscope. Images were analyzed using Fiji (ImageJ) software, and background fluorescence was removed using unstained controls to set the fluorescence brightness levels.

### 4.5 Transmission electron microscopy

DPM was added dry to Epon resin in an Eppendorf tube and polymerized in a 60°C oven for 24 h. Resin blocks were sectioned and mounted on grids as previously described.^36^ HSE samples were prepared for TEM as described previously.^36^ All grids were imaged using a Hitachi H7600 transmission electron microscope.

### 4.6 Scanning electron microscopy

DPM was adhered to silicon chips using a paintbrush. HSE were processed as for TEM to the point of dehydration and then were critical point dried (BAL‐TEC CPD 030, Leica) and attached to silicon chips. All chips were coated with 5 nm platinum using a Cressington Coating System 328 before imaging on a Hitachi S5200 SEM. TEM and SEM image measurements were taken using Fiji (ImageJ) software.

### 4.7 Cytokine analysis

One milliliter of media was harvested from the culture wells of models (inter‐experimental repeats were pooled) and frozen at −80°C. Samples were shipped to Eve Technologies (https://www.evetechnologies.com/) (Calgary, Canada) on dry ice for analysis on either the Human Cytokine Proinflammatory Focused 15‐Plex or Human Cytokine/Chemokine 48‐Plex Discovery Assay® Array.

### 4.8 Statistical analysis

Statistical significance was measured using a Student's *t*‐test, one‐way ANOVA with Dunnet's or Tukey's post‐test, or two‐way ANOVA with Bonferroni's correction using GraphPad Prism software.

## 5 CONCLUSIONS

In this study, we have outlined the development of a novel, immune‐competent human FT‐HSE containing MUTZ‐3‐derived CD1a+ and langerin+ LCs. We also demonstrated the ability of these models to mount a cytokine response to exposure to a chemical irritant and allergen that is greater than their non‐immune counterparts. Further to this, we demonstrated how DPM can also elicit a measurable inflammatory response in FT‐HSEs containing LCs, which demonstrates the importance of including immune cells when studying cutaneous inflammation. Further characterization of the DPM‐induced inflammatory profile of MUTZ‐3‐LC containing FT‐HSEs is required to improve our understanding of the immunological effects of air pollution on the skin.

## AUTHOR CONTRIBUTIONS

AS, TD, and SP contributed to the conception and design of the study. AS was responsible for the experimental work, with TD and SP providing supervision. AS wrote the first draft of the manuscript with input and editing from TD, and SP. All authors contributed to the article and approved the submitted version.

## FUNDING INFORMATION

This work was supported by funding from Procter & Gamble, Cincinnati, Ohio, United States, and UKRI Engineering and Physical Sciences Research Council EP/T022000/1 Physics of Life Network+ (PoLNet3).

## CONFLICT OF INTEREST STATEMENT

Author Stefan Przyborski collaborates and acts as a technical consultant for company Reprocell Europe Ltd. Teresa DiColandrea is a full‐time employee of Procter & Gamble (Cincinnati, OH, USA). Author Amy Simpson declares that the research was conducted in the absence of any commercial or financial relationships that could be construed as a potential conflict of interest.

## Supporting information


**Figure S1.** Additional electron microscopy images of skin equivalents. (a) (i) Transmission electron microscopy image of the uppermost layers of stratum corneum of an untreated skin equivalent demonstrating stratified, “brick and mortar” morphology. Note the different ultrastructure of the apical layer of stratum corneum, (ii) Full field of view image for Figure 1ai. (b) Full field of view transmission electron microscopy image for main text Figure 5ci. (c) Full field of view transmission electron microscopy image for main text Figure 5ci. (d) Full field of view scanning electron microscopy image for main text Figure 5di. (e) Full field of view scanning electron microscopy image for main text Figure 5dii. Scale bars: (a) 1 μm, (b, c) 0.5 μm, (d, e) 5 μm.

## Data Availability

The raw data supporting the conclusions of this article will be made available by the authors, without undue reservation.
