# Supplementary figures and images for: Assessing the impact of airborne particulate pollution on human skin utilizing a novel human skin equivalent containing MUTZ‐3‐derived Langerhans cells
Source: Bioeng Transl Med. 2024 Dec 13;10(2):e10738. doi: 10.1002/btm2.10738 (PMC11883110; doi:10.1002/btm2.10738)

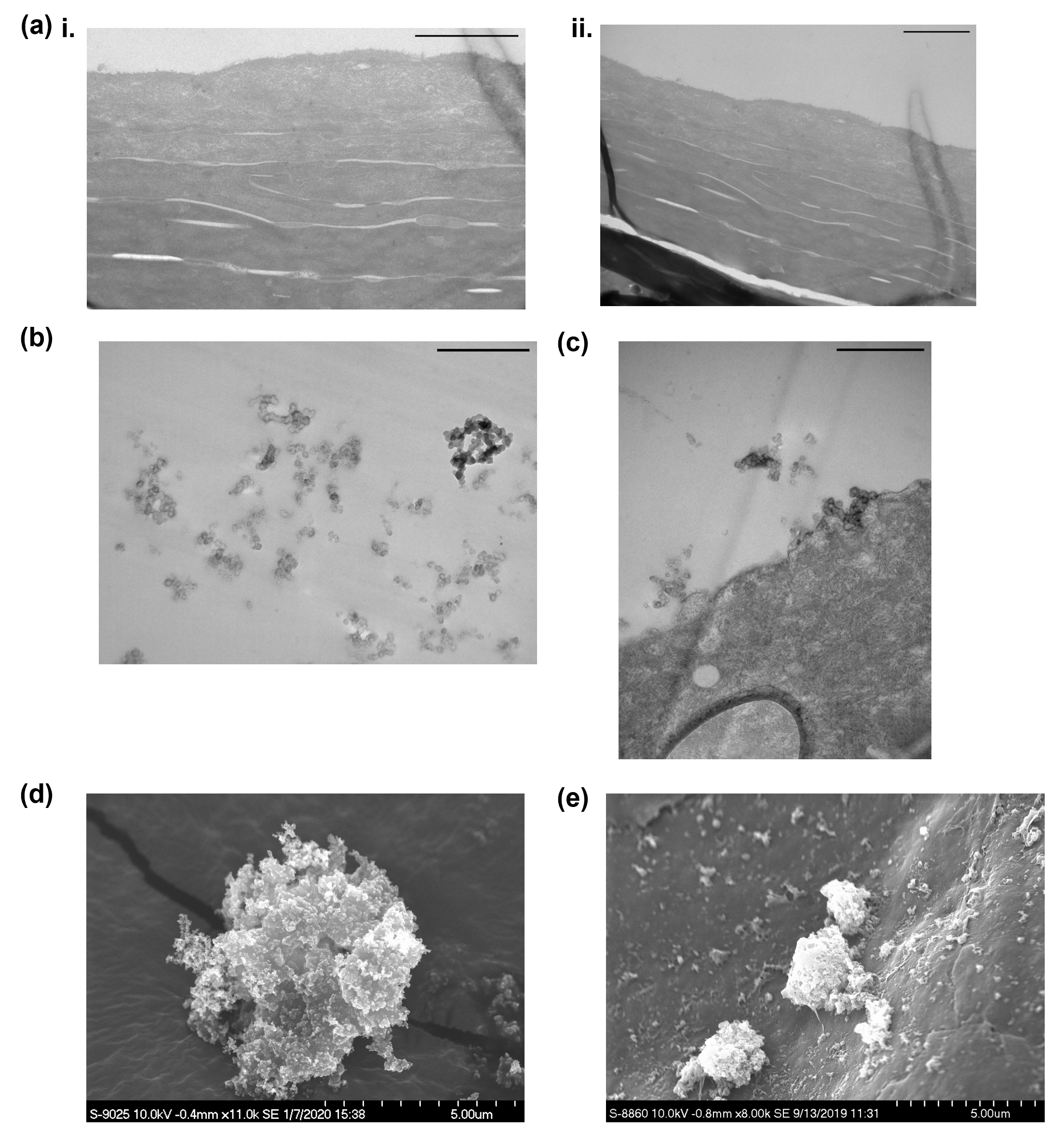

Supplement: Supplementary file 1 — Figure S1. Additional electron microscopy images of skin equivalents. (a) (i) Transmission electron microscopy image of the uppermost layers of stratum corneum of an untreated skin equivalent demonstrating stratified, “brick and mortar” morphology. Note the different ultrastructure of the apical layer of stratum corneum, (ii) Full field of view image for Figure 1ai. (b) Full field of view transmission electron microscopy image for main text Figure 5ci. (c) Full field of view transmission electron microscopy image for main text Figure 5ci. (d) Full field of view scanning electron microscopy image for main text Figure 5di. (e) Full field of view scanning electron microscopy image for main text Figure 5dii. Scale bars: (a) 1 μm, (b, c) 0.5 μm, (d, e) 5 μm. [file BTM2-10-e10738-s001.tif]
